# Surgical education and adult learning: Integrating theory into practice

**DOI:** 10.12688/f1000research.10870.1

**Published:** 2017-02-14

**Authors:** Prem Rashid

**Affiliations:** 1 Department of Urology, Port Macquarie Base Hospital, Rural Clinical School, The University of New South Wales, Port Macquarie, NSW, 2444, Australia

**Keywords:** surgical education, surgical training, adult learning, andragogy, pedagogy

## Abstract

Surgical education continues to evolve from the master-apprentice model. Newer methods of the process need to be used to manage the dual challenges of educating while providing safe surgical care. This requires integrating adult learning concepts into delivery of practical training and education in busy clinical environments. A narrative review aimed at outlining and integrating adult learning and surgical education theory was undertaken. Additionally, this information was used to relate the practical delivery of surgical training and education in day-to-day surgical practice. Concepts were sourced from reference material. Additional material was found using a PubMed search of the words: ‘surgical education theory’ and ‘adult learning theory medical’. This yielded 1351 abstracts, of which 43 articles with a focus on key concepts in adult education theory were used. Key papers were used to formulate structure and additional cross-referenced papers were included where appropriate. Current concepts within adult learning have a lot to offer when considering how to better deliver surgical education and training. Better integration of adult learning theory can be fruitful. Individual teaching surgical units need to rethink their paradigms and consider how each individual can contribute to the education experience. Up skilling courses for trainers can do much to improve the delivery of surgical education. Understanding adult learning concepts and integrating these into day-to-day teaching can be valuable.

## Introduction

It is important to understand the current concepts in surgical education in order to explore whether surgical training programs can meet contemporary theory. A change to any program needs to have, at least, a theoretical basis, although it is accepted that a theoretical basis does not always translate into practical reality. This article draws on adult education theory and argues that adult learning frameworks can offer ways to improve surgical education processes.

## Methodology

This article presents a narrative review, drawing on systematic review methods aimed at outlining adult education theory and how awareness of such conceptual theory can be related and integrated to the practical delivery of training and education in day-to-day surgical practice. Concepts were sourced from a reference text ^1^ and coursework materials. Additional material was found using a PubMed search of the words: ‘surgical education theory’ and ‘adult learning theory medical’. This yielded 1351 abstracts that offered either pure adult learning theory concepts and/or integration of theory into surgical education models. In total, 43 relevant articles with a focus on key concepts in adult education theory were used a core source material. Key papers were used to formulate structure and additional cross-referenced papers were included where appropriate.

## When did we learn to teach?

All graduating doctors engage in teaching as part of ongoing professional activity, and some do so without formally recognising it as such because it occurs in day-to-day practice, with colleagues, juniors, patients and ancillary staff. Learning to teach is not commonly part of any general medical curriculum. Like many professional endeavours, teaching by those more experienced becomes a matter of course. With the challenges of delivering clinical care and ensuring satisfactory educational experience, teaching in surgical education may need a more guided process.

Many surgeons and trainees alike equate surgical training with the technical aspects of the surgical craft, but it is known that there are a multitude of technical and non-technical skills that may be taught and learnt for true professional development ^2^.

It is understandable that trainers and trainees focus upon the technical aspects, since it is that aspect of the craft that differentiates it from other branches of medicine. Nevertheless, collaborative non-technical skills do play a significant role in day-to-day practice and, equally, need to be mastered in that context. Technical skills, including the ever-changing technological developments, require structured learning as well, to ensure acquisition of skill and patient safety. Failure to monitor these processes could lead to catastrophic consequences ^2– 4^. Therefore, the question is now not *if* the training process should change, but *how* it can change within the current system of delivering surgical training ^2^.

## Structured surgical education

Surgical education and training can be structured ^5^. The two terms have been used interchangeably, but clearly refer to different aspects of the global education process. Training refers to the practical aspects of learning the craft, and the education process encompasses the appreciation of the background complexities and knowledge ^5^. Both can include technical and non-technical aspects.

There are significant challenges in evaluating methods of delivering surgical education, including the technical and non-technical skills, and there continues to be a lack of correlation between the new teaching and assessment processes and their evaluation for efficacy ^2^.

Good teachers in any educational endeavour have a variety of common attributes, which includes a good knowledge base, experience, and an ability to patiently teach and empower students. In true educational reform, it is important to study innovative practices across other platforms and constantly review the methods of teaching, styles of delivery and, in doing so, develop curriculum and educational policy accordingly. Educational reform, however, will never be subject to randomised control trials because of the multitude of variables involved ^6^.

In addition, the surgical education model needs to be delivered in the context of the day-to-day provision of 24 hour surgical services. This key limitation is what most commonly explains the unusually long period of training required to produce a qualified surgeon. The apprenticeship model, with long immersion times, has been the traditional model, but is not efficient and continues to be improved.

In curriculum development, learning outcomes are important, and stepwise processes to achieve aspects of the curriculum need to be defined ^7^. Learning outcomes are about what the trainee should be able to do, or understand. However, there remain considerable gaps between the documented curriculum, how it is then taught and experienced, and subsequently assessed ^6^. Innovation and managing logistics are key to finding more effective methods ^8^.

## Stepwise learning

In an ideal world, stepwise training processes will lead to global proficiency in a professional activity ^9– 12^. Close supervision will ensure patient safety. It is interesting to reflect that the ‘conservative legacy’ of surgery has also been an impediment in terms of surgical education ^13^.

There are critical educational issues that need to be reviewed to address surgical education. These are:

1. improved working conditions and reduction of hours in the working day;
2. the apprenticeship model with a prolonged time-based system and ‘learning by osmosis’ needs to be modified;
3. the need to embrace contemporary reflective learning;
4. the need to structure technical and non-technical objectives in surgery;
5. the need for surgical educators, as well as on-site conditions, to provide surgical teaching;
6. the need to address professionalism to meet profession and community expectations.

Surgical teaching units need to rethink their paradigms and consider how *each* individual, within a unit, can contribute to the education experience. Up-skilling courses can do much to improve the delivery of surgical education.

## Evolving the teaching model

We still foster a master-apprentice system, although more structure with defined objectives have become the norm, as this concept fits in with contemporary, adult learning principles. Our trainees do ‘learn on the job’ as a matter of course. Much of the original model was unstructured, and that has progressively changed so that objectives have become clearer and assessment processes have become more transparent and fair, including being subject to query and appeal. As the system has evolved, the greatest change has been in assisting busy clinical surgeons, who often teach ‘pro-bono’, to up-skill and adopt progressive methods.

## What has changed?

• Surgical training programs have evolved over time including **objective assessment processes**, with **constructive feedback** being the mechanism for dealing with performance issues. Assessment is about a process to establish if a pre-determined standard has been achieved in a competency.
• The process of assessment has become more **practical and workable** within the **time and resource constraints of a busy clinical unit**.
• The process is also **more clearly outlined, reasonable, valid, objective, fair and reliable**.
• Providing **constructive, objective and specific feedback,** as well as an opportunity to discuss areas that require attention, has provided scope for improvement. There should be a clear appreciation that failure to correct at an acceptable rate should have consequences that are outlined ^14^.
• All objectives that are set, need to be relevant, achievable, specific, timely (for the level of training), regular and stimulate the desired learning ^15^.

Assessment processes have evolved over time and now include a variety of technical and non-technical competency assessments in line with key competencies *(e.g., http://www.surgeons.org/becoming-a-surgeon/surgical-education-training/competencies/).* Trainees are also often given the opportunity to provide confidential assessment of the training post at the end of each term. This is primarily used to assist in accreditation processes.

## How can we improve teaching?

There has generally been a chasm between educational theory and practice, which is not unique to medical education. Theory may have practical flaws, but often does inform and improve practical day-to-day performance ^16^.

## Adult learning principles

There are a number of theories to help us improve the way we understand medical education.

• ***Adult learning characteristics*** ^17^ – differences between adult and child learners
• ***Adult’s life situation*** ^18^ – where individuals are in the variable stages of life
• ***Changes in consciousness*** ^19^ – ability to reflect upon experience and environment ^20^.

No single theory can be applied universally. Knowles’ *Andragogy* concept was proposed as the ‘ *art and science of helping adults learn*’ ^21^. This concept was modified to describe it as a *continuum* from *pedagogical* principles. This concept ultimately is about what is unique to adult learning and, as such, different to childhood learning ^22^. The context in which adults learn is different because they most often *choose* to learn, which is a different motivation to compulsory schooling. The adult learner also has a large social, and often, professional experience, which can contribute to reflection. The pace and meaning for an adult who pursues further education is also different, as are the pressures of life in balancing work and personal matters ^21, 23, 24^.

## Theories of adult learning

There are a number of theories that guide us in appreciating adult learning concepts, and understanding these can help us shape teaching programs. These include:

### Social cognitive theory

Social cognitive theory acknowledges the interactive or social aspect of learning, and the influence of the environment also plays an important part. The collective is made up of the environment, personal factors and behavioural issues ^25^. Feedback becomes a major influence as adults take on new skills. Adults have the ability to apply forethought, bring experience and can self-reflect. They can regulate as they integrate new information. This means that adult learning must include objectives that are desired and relevant, task activities that lead to fulfilment of the desired knowledge and an ability to develop upon what they bring to the learning environment ^20^.

### Reflective practise

Reflective practise is one of the core concepts of adult professional development where new information is interpreted in the light of past knowledge and experience. Adults will rework and reframe from the perspectives they have ^26^. Education principles need to encourage thinking before, during and after the event ( *reflection pre-action*, *reflection in-action* and *reflection on-action*) ^27^.

### Transformative learning

Transformative learning is the process by which we internalise and interpret information based on our own experiences to date. We bring an existing ‘way of thinking’ that is used in learning ^28^. The transformation is the evolution of the paradigm based on the learning during the process. Encouraging social discourse, group participation and evaluation of individual differences, is all part of this process. Assumptions are questioned and the consciousness is elevated to a new point of contextual self-appreciation. Adult learners embrace transformation if they feel that the goals are worth attaining and they have control over the learning process ^29, 30^.

### Self-directed learning

Self-directed learning has become part of professional best practice in maintenance of standards. The premise of this is self-motivation, self-direction and self-management. Much of the knowledge is context-dependent and subject to a self-assessment of needs. Multiple portals of easy-to-access and flexible educational delivery platforms are important to make this effective. Having frequent and clear assessment methods help to maintain focus and effectiveness. The student learns more when they are given control of the ‘what?, when?, how?’, and possibly the most important for them, the ‘why?’ ^31– 33^

### Experiential learning

Experiential learning, developed by Kolb ^34^ needs to be adopted more formally with structured stages of the learning cycle, which include:

▪ performing a task;
▪ reflecting upon that task with input from others;
▪ identifying areas where improvement could be made; and
▪ readjusting in some way before a new cycle.

Feedback, reflection and debriefing could occur during, or after the event, or both ^35^. This could then be linked into the learning outcomes outlined in the curriculum. Trainees need to know:

▪ what they were expected to learn;
▪ how that could occur;
▪ how it would be assessed; and
▪ how it would become useful in their professional lives.

The teacher does assume the role of the taskmaster and expert who provides guidelines and is charged with monitoring the process ^36^.

### Situated learning

This is where learners participate in community activities and there is a socio-cultural basis to the process. There is a purpose and connection within a historical context and learning occurs via social interactions and developing relationships that are contextual within the culture. Learners develop an understanding of ‘the way it is done’. This is part of the ‘hidden curriculum’ in medical practice, the values and judgements that help one ‘belong’ to a community, without which one can feel isolated. Role-modelling helps demonstrate some of the social interactions ^37^.

### Learning in communities of practice

This involves a ‘trajectory’ beyond situated learning. This is where learners move from peripheral participation into full participation, embracing all the values and experiences within the community. Here learners already ‘belong’ and develop further understanding. They begin to appreciate how the community interacts with the ‘outside’ world. In medical education, we begin with a theoretical knowledge base, (ideally) progress to using simulated experiences, then start to observe real-world activity before beginning the immersive experience of becoming part of that world and growing professionally within that environment ^38^.

## Application of theory

Adults need to feel supported within their education framework and they must be allowed to bring in their own know-how and develop with graduated skill levels. It is important that they be given clear direction in terms of what the standards and objectives are, so, assuming they are motivated, they can be allowed to self-drive and manage their own learning. Learner autonomy is important in this context.

## An example: Operative surgery

This can be brought together in an example of a supervisor sitting in on an operative case with a surgical trainee, where the three recognised phases of an operation will be broadly covered. One of the key issues is for the supervisor to be available, which means to be in the environment, if for no other reason than to give advice, if required, and ensure that things go smoothly. Adult learners need to know that they have the supervisor’s confidence and support and that they will be allowed to stretch their skillset to try to solve any problems they may encounter, before the supervisor steps in.

### Pre-operative assessment

The supervisor and trainee:

• discuss the **case, history, examination and investigations to date** (being clear on how the patient came to be in the procedural environment and assessing the needs of the patient, learner and teacher). Good questions for the trainer to ask are, “ *X, you have done Y number of these, what aspects do you feel you need to improve upon?*” or “C *an you outline what issues are worth covering with the patient when discussing this operation in this setting?*” or “W *hat do you want to focus on today?*”
• discuss the **steps of the operation** and **who will undertake which part** – the supervisor should *not set unexpectedly-high standards, only what is reasonable to achieve for the level of the trainee.*
• consider the ‘ **what ifs?’** – issues that may come up and how to manage them.
• consider what **equipment** and **contingencies** need to be in place.
• outline **what is planned**, discuss **informed consent** issues – these enquiries of objectives and aims can give insight into the trainee’s self-assessment ability.
• undertake the ‘ **time out’** process correctly.

### Intra-operative teaching

• The trainee tries to follow the **pre-operative plan** and, if there is a **deviation**, ensures that all are aware of the reason why.
• The supervisor guides through the **focal technical steps**.
• The supervisor gives **immediate feedback** on what is being done well and what could be done differently.
• The supervisor considers **allowing the trainee to do more** if they are progressing well and advises them accordingly.
• The supervisor **watches for over-confidence** and guides the trainee back if required.
• The supervisor must **try not to ‘take over’,** unless necessary. It is better to talk the situation through and let the trainee do what they can for themselves *(taking over can be confused for condemnation or lack of patience – both of which may exist).*

### Post-operative debriefing

This can start to occur as the trainee is bringing the case to a close, but some trainees may find this distracting, which may hamper their performance.

The supervisor should:

• plan to debrief in a quiet **environment** (not always possible in a busy surgical service).
• ask the trainee **how they think they went**, stimulate reflection and teach general rules; help them review their performance; explore ways they can set some goals to help address deficiencies, e.g., “ *Should we look at ways to help you with this issue?*” or “ *What do you think about addressing smaller chunks of the main task and then we can set some methods of getting those nailed in X (period of time)?*”
• provide **specific examples** of how the trainee could have performed better, e.g., “ *When you got to point X and did Y, I think doing Z may have helped you progress better.*” This gives the trainee an idea of what the supervisor would have done.
• **congratulate the trainee** on what they have achieved, e.g., “ *I think you did that better this time because the way you did X worked much better and I could see that you were getting that aspect.*” A supervisor may think the trainee did well, but if they don’t communicate this, the trainee may feel that some aspect was not done well. Supportive communication is important.

Vollmer, *et al*. offer some relevant issues to consider in this context: ^39^

• **Trainee preparedness** (they need to know whether their level of preparedness met the supervisor’s expectations.) If that hasn’t happened, then it is important to ask why. The crucial aspect of this is to genuinely enquire rather than using an accusatory tone. Trainees need help to establish GOALS via the SMART process – keeping the process manageable. (SMART goals are specific, measureable, action-oriented, realistic and time-bound.) Trainees need to appreciate the requirement for the smaller, intrinsic goals within the greater concept of what they are trying to ultimately achieve.
• **Quality and style of communication** (supervisors need to be supportive, clear and compassionate).
• **Time constraints** (relevant in most clinical units). This means that debriefing may be difficult to organise and needs forethought. There must be a plan to deal with in-service time constraints and, if necessary, find another moment more suitable to the task.
• **Environment** (may be difficult to achieve the ‘quiet room’ ideal).
• **Teacher engagement** (most clinical teachers were never taught to teach!). It needs to be understood that each teacher must up-skill along the way and develop in their role.
• **Patience and tolerance** (a skill that can take time and usually comes with clinical experience and progressive self confidence).
• **Autonomy** (whether the trainee feels like they were capable and allowed to do what they could – lack of insight notwithstanding).
• **Feedback** (preferably immediate and constructive as reprimands rarely achieve the perceived desired objectives). For example, “ *What did you do well? What could you have done better? What areas do you need help with? How can I help you in the areas you are yet to master?”*
• **Ethical or legal issues** that may have come up during the case. These can be complex and need exploring, as this may be the only opportunity for experience. They could be raised at a later time *, e.g., “With regard to Mrs Smith the other day, what did you think of what happened?”* This could apply to an adverse event that they may have caused or witnessed. These types of issues are critical for discussion, as they do not appear in textbooks.

Feedback for adult learners differs in a number of ways: ^40^

• This can begin with **self-assessment**. Most adult learners have an idea of how they are performing. Some may be too hard on themselves, but that can be tempered with balanced discussion. The key is to **listen** and become **aware** of how they perceive they are performing. **Help them reframe** their perspective if it seems too harsh.
• It is easy to ramble sometimes, but it is most important to **focus on the main objectives** and **be specific** with examples.
• It should be **immediate** (or soon after the event) – bringing up past events can be confusing and unfair.
• It should be **conceptually relevant** – about what just happened.
• It should be **constructive** – the general principle is to instil confidence.
• It should be **compassionate and empathetic** – putting themselves in the trainee’s shoes.
• Plan to **observe rather than judge,** using comments like,
  “ *I noticed …*” or “ *I observed …*”
• **Debriefing** consolidates learning.
Consider **emotional state** – may need to delay feedback, or when serious, consider employee assistance programs.

Theoretical frameworks help construct ways to adapt learning programs, in summary they include: ^20^

• Viewing the learner as a central active contributor.
• That the total experience is more important than individual aspects.
• Learning is related to solutions and understanding real-life issues.
• Past and inter-current social experiences play a key role in framing new knowledge.
• Self-awareness, attitudes and beliefs are important for contextual development.
• Individuals can usually self-regulate and manage their learning, and are commonly motivated to do so.
• Self-reflection is central to the learning experience. The picture becomes more complete when there is a blend of theory, coupled with new information and past, personal and professional experiences.

In addition, there are characteristics that help define teachers who are seen to be more engaged. They include being:

• available and approachable;
• understanding, and using fair performance indicators;
• non-judgemental;
• capable (to help take over and teach the next steps); and
• engaging of the whole team.

Further positive features include:

• The **quality of the relationships** formed are the most important factor in the effectiveness of clinical supervision, more than the method of supervision used ^41^.
• Much of the **early theory** has come from nursing, psychology, teaching and social work literature. Current supervision and teaching practice has only recently developed theoretical basis ^41^.
• Supervision and teaching is **complex**, involving changing clinical situations and ensuring that patient safety and care is preserved.
• Trainees and trainers can have widely **disparate views** of the clinical teaching encounter ^39^. Good communication remains the key.

## Challenges ahead

It can be easy to offer theoretical discourse on surgical education, but equally it must be acknowledged that translation into practice and proof that the translation has met the desired objectives is where the real challenge lies. Simulation in surgery, for example, is still not where it is in the aviation industry, where certification is possible with an assessment of performance in multiple scenarios, yet it remains a desired objective ^42– 46^. The objective assessment of surgical technical and non-technical skill continues to challenge educators, who need to be able to teach in a step-wise fashion and then ‘sign-off’ a skill base. These tools do exist, as outlined, but the practical implementation by busy clinicians remains the key ^47^. Ultimately, there must be a desire by surgical supervisors to adopt and implement educational reform and systematically measure the effectiveness along the way. Each environment will present unique situational challenges that will need to be adapted to ensure efficiency of clinical services, patient safety and achievement of educational objectives.

## Conclusions

The key messages for teaching, supervising and assessing are objectivity, transparency, fairness, empathy, support and compassion. Focus on upskilling of surgical supervisors will help deliver a better education framework. While some of the theoretical basis may be challenging to prove, it is clear that, despite there being uncertainty in complex teaching situations, the basics will often help bring it together, and that very process will continue to help in the next encounter, as constant reflection and assessment lead to integration and growth.
