# Supplementary material for: Surgical education and adult learning: Integrating theory into practice
Source: F1000Res. 2017 Feb 14;6:143. [Version 1] doi: 10.12688/f1000research.10870.1 (PMC5345767; doi:10.12688/f1000research.10870.1)
Supplement: Peer review history

# Peer review: Referee response for version 1

**DOI:** 10.5256/f1000research.11720.r20806

**Published:** 2017-03-08

**Authors:** Matthew Winter

**Affiliations:** 1 Gosford District Hospital, Gosford, NSW, Australia

A very useful contribution to the literature to engage both supervisors and trainees alike. This paper addresses the importance of critically analysing our approach to surgical education and training while focusing on the theories of adult learning to guide such approach.

 Title is very appropriate and abstract summarises article sufficiently.

 Design of article is appropriate and in keeping with its attempt to summarise the existing theories of

 adult education and provides an excellent narrative of extending these theories into the context of

 operative surgery. The opinions presented are well constructed, balanced and based on foundation

 literature. Very well done.

 I make the following suggestions to improve the article.

 • Adult learning principles (page 3) are referred to as theories under its heading. Two paragraphs later the heading Theories of adult learning surfaces. This created a small degree of confusion. Could this be addressed to simplify reader dissonance.

 • The theory of “Experimental learning” seems similar to “Reflective learning”. Does the author feel that separating these into two theories enhances the paper?

 • The use of dot points throughout the paper is inconsistent. Especially the word following the dot point – sometimes listed as a capital letter sometimes not.

 • The term ‘Up-skilling’ is spelt differing ways throughout the article.

I have read this submission. I believe that I have an appropriate level of expertise to confirm that it is of an acceptable scientific standard.

# Peer review: Referee response for version 1

**DOI:** 10.5256/f1000research.11720.r20746

**Published:** 2017-03-07

**Authors:** Mohan Arianayagam

**Affiliations:** 1 Nepean Private Hospital, Sydney, NSW, Australia

This is a well written and well researched piece on surgical education. The article reviews current educational theory which is certainly very useful for a surgical audience and also highlights the challenges of teaching in a busy surgical service and postgraduate environment. The model of surgical teaching has been unchanged for decades with the teacher/apprentice model being the mainstay of teaching. While most of us are keen to teach we are not trained as teachers. This article helps provide a framework for teaching in this environment, including techniques for feedback to “close the loop”.

 Overall a very useful article.

I have read this submission. I believe that I have an appropriate level of expertise to confirm that it is of an acceptable scientific standard.

# Peer review: Referee response for version 1

**DOI:** 10.5256/f1000research.11720.r20600

**Published:** 2017-02-28

**Authors:** Kathryn Rzetelski-West

**Affiliations:** 1 Department of Urology, West Coast Urology, Geelong, Vic, Australia

This opinion article is a very interesting piece in the context of the current surgical training climate in Australia and around the world. With reduced working hours, emphasis on operating room efficiency, increased complexity of surgical cases, and greater emphasis of medico-legal implications; surgical education needs to be improved.  The author attempts to give a summary on the educational theory behind adult learning and integrate theory into practical advice for clinicians. This is important, as most surgical teachers have no academic education training or background, despite this important job.

 The article does contain all the components one would expect from a journal article, although one wonders whether a PubMed search of ‘surgical education theory’ and ‘adult learning theory medical’, reminiscent of a metanalysis, was necessary in an opinion article.

 The title and abstract are appropriate. The article is well organised. The flow of the article is logical with the theoretical basis presented first, followed by the practical implementation into surgical practice. The article is an excellent review of adult learning theory and its varying components. The author does a good job at synthesizing the literature. The differing components comprising adult learning theories are brief but informative, and do not overwhelm the reader in complexity. The references used are appropriate for any readers wanting more in-depth reading. The article is well written and easy to understand. The example of operative surgery is, in particular, very easy to read and will appeal to clinicians. The dot point format aids its readability, as does the practical nature of the advice.  The structure of pre-operative assessment, intra-op teaching, feedback and post-operative debriefing is particularly effective.

 Possible areas for consideration:

1. Structured surgical education – The first sentence in this paragraph feels disjointed and fragmented. It does not seem to relate to the following sentences. It may improve sentence flow if it specified which aspects of structured surgical education and training it was referring to e.g. its objectives, assessment etc.
2. Under the paragraph of intra-operative teaching, the author discusses allowing the trainee to do more (dot point 4) and the supervisor must try not to take over (dot point 6). This is reminiscent of the theories of ‘Vygotsky and the Zone of Proximal Development’ ^1^ and ‘scaffolding’ ^2^. Vygotsky describes what a learner can do with the help of a more capable other ^1^, and scaffolding ^2^ refers to where it is as important for teachers to know when to step back from supporting learners as it is to provide that support. Although not strictly based on adult learning theory, they are relevant to the ways in which adults learn. A major criticism of adult learning principles is that adults actually learn in similar ways to children ^3^ ^4^.
3. It may be prudent to note that although adult learning theory is very popular, it is a controversial theory with opponents as well as supporters. There are educationalists who believe that the learning style of adults are essentially the same as children. There is only weak research supporting adult learning theory, and much of the research has been shown to be inconclusive and have contradictory outcomes. Indeed Knowles later stated: “I am at the point now of seeing that andragogy is simply another model of assumptions about learners to be used alongside the pedagogical model of assumptions, thereby providing two alternative models for testing out the assumption as to their 'fit' with particular situations. Furthermore, the models are probably most useful when seen not as dichotomous but rather as two ends of a spectrum, with a realistic assumption in a given situation falling in between the two ends” ^5^.
4. Up-skilling of surgical supervisors is one of the major solutions offered in this opinion article, however little practical advice of what exactly this should consist of is not detailed. It may be beneficial to offer more detailed advice on what skills should be improved and how they should be obtained.
5. Up skilling is spelt three different ways in the article: up skilling, up-skilling and upskilling.

I have read this submission. I believe that I have an appropriate level of expertise to confirm that it is of an acceptable scientific standard.

## References

[1] VygotskyL: Mind in society: The development of higher psychological processes. *MA: Harvard University Press*.1978;

[2] WoodDBrunerJRossG: THE ROLE OF TUTORING IN PROBLEM SOLVING. *Journal of Child Psychology and Psychiatry*.1976;17(2) : 10.1111/j.1469-7610.1976.tb00381.x 89-100 10.1111/j.1469-7610.1976.tb00381.x 932126

[3] DavenportJDavenportJA: A chronology and analysis of the andragogy debate. *Adult Educational Quarterly*.1985;35(3) :

[4] HouleCO: The design of education. *Jossey-Bass*.1972;

[5] KnowlesMS: The modern practice of adult education: Andragogy versus pedagogy (Revised edition). *Englewood Cliffs: Prentice Hall/Cambridge*.1988;
